# Supplementary material for: The Role of Negative Perfectionism and the Relationship between Critical Thinking and the Halo Effect: Insights from Corporate Managers in Human Resources
Source: Behav Sci (Basel). 2023 Jun 26;13(7):533. doi: 10.3390/bs13070533 (PMC10376162; doi:10.3390/bs13070533)
Supplement: Supplementary file 1 [file behavsci-13-00533-s001.zip › behavsci-2450566-supplementary.pdf]

Supplementary Materials

1. Chinese Critical Thinking Scale

Original version (used in our research)

1. 与不同文化的人交往是一件有趣的事情。
2. 除了我从事的工作方向外，日常生活中我也会进行其他方面的阅读活动。
3. 我没有办法同与自己观念相差很大的人进行很好的交流。
4. 平时我经常与别人分享对事物的观点。
5. 我并不经常与别人谈论我对某个事物的看法。
6. 与其与别的人在一起，我更喜欢独处。
7. 一旦开始着实做某事，除我正在用的方法外，我不会考虑其他可能的解决问题的方法。
8. 在现实生活中，我会尝试从别人的角度来看待同一问题。
9. 我在解决现实生活中的问题时，经常有其他的替代方案。
10. 既然我做出了决定，我就不会反复考虑其他选择。
11. 对于一个话题，当我与别人产生意见分歧时，会影响我与别人的交流。
12. 我在做出决策时经常考虑父母长辈的意见，因为他们会真正地关心我。
13. 在日常生活中，我经常提出问题并试图寻找答案。
14. 我喜欢出去了解事物是如何运作的。
15. 我认同“存在必有合理性”这句话。
16. 我不会怀疑大家公认的事情。
17. 我的好奇心往往得不到别人的欣赏。
18. 同龄人面对难以决断的问题时喜欢找我商量。
19. 社会地位较高的人的视野更开阔，所以其观点相对更可信。
20. 对我而言，尝试解决富有挑战性的任务是种乐趣。
21. 处理复杂的问题时，我往往无从下手。
22. 一般做事情之前，我清楚地知道自己的目的地何在。
23. 在我看来，考试主要是考察对于知识的记忆，所以背诵是我学习的主要手段。
24. 课堂上的学习与日常生活中的运用是两码事。
25. 周围的人往往认为我做事情容易冲动，但我认为敢想敢做是件好事。
26. 为了解决问题，我会同时收集不同观点的信息进行整理比较。
27. 我总是会先分析问题的重点所在，然后才解决它。
28. 我善于有条理地按照一定步骤解决问题。
29. 当我看到一个新产品的说明书复杂难操作时，我倾向于放弃阅读。
30. 我能够区分生活事件中那些事情是重要的。

Translated English version

1. It is interesting to interact with people from different cultures.
2. I partake in other reading activities in my daily life in addition to at work.
3. I don't have the ability to communicate well with people whose ideas are very different from my own.
4. I usually share my views on things with others.
5. I don't often talk with others about my opinion about something.
6. I prefer to be alone rather than with other people.
7. Once I find a way to solve a real-life problem, I don't consider other possible ways to solve the problem other than the one I am using.
8. In real life, I try to see the same problem from someone else's point of view.
9. I often consider alternative strategies when solving real-life problems.
10. When I make a decision, I do not reconsider alternatives.
11. When I disagree with others about a topic, it affects my communication with others.
12. I often consider the opinions of my parents' elders when making decisions because they would truly care about me.
13. In my daily life, I often ask questions and try to find answers.
14. I like to go out and learn how things work.
15. I agree with the saying "there must be a reason for existence".
16. I don't doubt what is accepted.
17. My curiosity is often not appreciated by others.
18. My peers like to talk to me when they are faced with a problem that is difficult to decide.
19. People of a higher social status have a broader perspective, so their views are relatively more credible.
20. It is fun for me to try to solve challenging tasks.
21. When dealing with complex problems, I am often at a loss.
22. I usually know exactly where I'm going before I do something.
23. In my opinion, exams mainly involve recalling memorized knowledge, so memorization is my main means of studying.
24. Learning things in the classroom and applying them to daily life are two different things.
25. People around me tend to think that I am impulsive, but I think it is good to think and act boldly.
26. In order to solve a problem, I will collect information from different points of view at the same time to organize and compare.

27. I will always analyze the focus of a problem before solving it.
28. I am good at solving problems in a structured way and following certain steps.
29. I tend to give up reading the instructions of a new product when I see that it is complicated and difficult to operate.
30. I can distinguish those things that are important in life events.

2. Multidimensional Perfectionism Scale

Original version

1. My parents set very high standards for me.
2. Organization is very important to me.
3. As a child, I was punished for if things were less than perfect.
4. If I do not set the highest standards for myself, I am likely to end up a second-rate person.
5. My parents never tried to understand my mistakes.
6. It is important to me that I be thoroughly competent in everything I do.
7. I am a neat person.
8. I try to be an organized person.
9. If I fail at work/school, I am a failure as a person.
10. I should be upset if I make a mistake.
11. My parents wanted me to be the best at everything.
12. I set higher goals than most people.
13. If someone does a task at work/school better than I, then I feel like I failed the whole task.
14. If I fail partly, it is as bad as being a complete failure.
15. Only outstanding performance is good enough in my family.
16. I am very good at focusing my efforts on attaining a goal.
17. Even when I do something very carefully, I often feel that it is not quite right.
18. I hate being less than the best at things.
19. I have extremely high goals.
20. My parents expect excellence from me.
21. People will probably think less of me if I make a mistake.
22. I never felt like I could meet my parents' expectations.
23. If I do not do as well as other people, it means I am an inferior human being.
24. Other people seem to accept lower standards from themselves than I do.
25. If I do not do well all the time, people will not respect me.
26. My parents have always had higher expectations for my future than I have.
27. I try to be a neat person.
28. I usually have doubts about the simple everyday things I do.
29. Neatness is very important to me.
30. I expect higher performance in my daily tasks than most people.

Translated Chinese version (used in our research)

1．我的父母曾给我定下很高的标准。
2．做事有条理有系统对我是十分重要的。
3．孩童时，我曾因做事欠完满而受到惩罚。
4．如果我不给自己定下最高的标准，我很可能沦为次等的人。
5．我的父母从来都不试着去了解我的过错。
6．我必须完全胜任自己所做的每一件事，这对我来说至关重要。
7．我是个整洁的人。
8．我尽量做一个有条理的人。
9．如果我在工作/学校中失败，这说明我整个是一个失败的人。
10．我若犯了错误就应该感到烦恼不安。
11．我的父母曾希望我在各方面都是最出色的。
12．比起大多数人，我定下更高的目标。
13．若有人在工作或学习比我强，我会觉得自己整个地失败了。
14．做事或学习的时候若是有部分的失败，我会觉得自己完全失败了。
15．在我的家里，只有出色的表现才算够好。
16．我善于集中精力以达到自己的目标。
17．尽管我小心翼翼地做事，还是经常感到自己做得不太正确。
18．我厌恶做事不能做得最佳。
19．我有极高的目标。
20．我的父母曾经期望我做得特别出色。
21．假如我犯错误，人们很可能会轻看我。
22．我从不觉得自己能够满足父母对我的期望。
23．如果我不能做得跟别人一样好，说明我是个低人一等的人。
24．比起我，别人似乎比我更能接受低一些的标准。
25．如果我不能始终表现出色，我就会失去别人对我的尊敬。
26．比起我，我父母对我的将来经常有较高的期望。
27．我尽力成为一个整洁的人。
28．我经常对一些日常小事也犹豫不决。
29．整洁对我来说是十分重要的。
30．比起大多数人，我要求自己在每天的工作中有更好的成绩。
31．我是一个有条理的人。
32．我的工作进度缓慢，因为我常重复那些工作。
33．为了把一件事情做好，我需要花较长的时间。
34．我犯的错越少，喜爱我的人就越多。
35．我从不觉得自己能达到我父母为我定下的标准。

3. Halo Effect

Chinese version (used in our research)

1. 面相宽厚、举止得体的人比较忠诚可靠。
2. 王经理的团队业绩突出，说明他领导有方，德才兼备。
3. 工作绩效好的员工擅长处理人际关系。
4. 爱整洁的人做事情有条理。
5. 有良好教育背景的人学习能力强，工作绩效更好。

English version

1. People with a broad face and decent manners are more loyal and reliable.
2. People with a good educational background are better learners and perform better at work.
3. The outstanding performance of a manager's team shows that the manager is a competent leader with both talent and moral integrity.
4. An employee whose job performance is high is also good at dealing with interpersonal issues.
5. Neat people are also well-organized.
